# Endoscopic and direct vision approaches in minimally-invasive mitral and tricuspid valve surgery – insights from the mini-mitral registry

**DOI:** 10.1186/s13019-025-03742-x

**Published:** 2025-11-22

**Authors:** Torsten Doenst, Paolo Berretta, Tom C. Nguyen, Nikolaos Bonaros, Marc Gerdisch, Mauro Rinaldi, Joerg Kempfert, Joseph Lamelas, Frank Van Praet, Tristan Yan, Loris Salvador, Antonios Pitsis, Manuel Wilbring, Davide Pacini, Antonio Fiore, Nguyen Hoang Dinh, Pierluigi Stefano, Hristo Kirov, Marco Di Eusanio

**Affiliations:** 1 Department of Cardiothoracic Surgery, Friedrich-Schiller-University Jena, University Hospital, Jena, Germany; 2 Cardiac Surgery Unit, Lancisi Cardiovascular Center, Polytechnic University of Marche, Ancona, Italy; 3 Baptist Health Miami Cardiac & Vascular Institute, Miami, FL USA; 4 Department of Cardiac Surgery, Medical University of Innsbruck, Innsbruck, Austria; 5 Franciscan Health Indianapolis, Indianapolis, IN USA; 6 Cardiac Surgery Unit, University of Turin, Turin, Italy; 7 Department of Cardiothoracic and Vascular Surgery, German Heart Center Berlin, Berlin, Germany; 8 Division of Cardiothoracic Surgery, University of Miami, Miami, USA; 9 Cardiac Surgery Department, Hartcentrum OLV Aalst, Aalst, Belgium; 10 Department of Cardiothoracic Surgery, The Royal Prince Alfred Hospital, Sydney, Australia; 11 Division of Cardiac Surgery, S. Bortolo Hospital, Vicenza, Italy; 12 Cardiac Surgery Department, European Interbalkan Medical Center, Thessaloniki, Greece; 13 Center for Minimally Invasive Cardiac Surgery, University Heart Center Dresden, Dresden, Germany; 14 Cardiac Surgery Department, Sant’Orsola Malpighi Hospital, University of Bologna, Bologna, Italy; 15 Department of Cardiac Surgery, Hôpitaux Universitaires Henri Mondor, Assistance Publique-Hôpitaux de Paris, Créteil, France; 16 University of Medicine and Pharmacy, Ho Chi Minh City, Viet Nam; 17 Cardiac Surgery Unit, Careggi University Hospital, Firenze, Italy

**Keywords:** Mitral valve, Tricuspid valve, Minimally-invasive surgery, Endoscopic surgery

## Abstract

**Background:**

We investigated the international mini-mitral registry (MMIR) for differences in minimally-invasive access for surgery on the mitral and tricuspid valve. We compared direct vision with partially or fully endoscopic approaches.

**Methods:**

From 2015 to 2021, 7,513 consecutive patients underwent mini-MVR ± TVR in 17 international Heart-Valve-Centers. Data were collected according to MVARC definitions and 6463 patients undergoing first time mitral with or without tricuspid valve surgery were analyzed. Uni- and multivariable regression analyzes were performed to compare the different approaches.

**Results:**

Patients were 65 years (57% male) and oldest in the direct-vision group (*n* = 1594). Endoscopes (video-assisted: *n* = 2850, fully-endoscopic: *n* = 1963) were used in slightly more selected patients (less obesity, diabetes, dialysis, CAD, pulmonary hypertension, reduced LVEF and urgent status compared to direct vision). Robot was used in 56 cases (most selected, no mortality, not further analyzed). Fully-endoscopically, most cases were repairs, concomitant tricuspid surgery was lowest (13% vs. 20%) and both cardiopulmonary bypass and cross-clamp times were longest (90 min, IQR 71–113 min). Cross-clamp times were shortest in the direct vision group (-20 min). Technical success was high (above 96%), in-hospital mortality and stroke rates low and not significantly different between groups. Low output was highest with direct vision and acute kidney injury highest fully-endoscopically. However, this difference was not significant.

**Conclusions:**

In this large registry, the type of minimally-invasive approach did not significantly affect outcome. It appears that fully endoscopic and robotic cases are used more selectively. Mastering both techniques may optimize patient care.

## Background

Minimally-invasive access has become routine for mitral and tricuspid valve surgery. However there is great debate, whether adding an endoscope to procedures that may be performed through direct vision improves the surgeon’s ability to perform the procedure and therefore improve outcomes [1–3]. Variations of endoscope use are subdivided into video-assisted, fully endoscopic and robotic procedures. A direct comparison of any of the strategies in a randomized trial has never been performed. The Mini-Mitral-Registry (MMIR) is the largest summary of expertise in minimally-invasive mitral surgery worldwide. We therefore exploited this registry to address the question whether endoscopic support for minimally-invasive mitral surgery affects outcomes.

## Patients and methods

The MMIR is an independent registry involving 17 international Heart Valve Centers. The rationale and methods of MMIR were previously reported [4]. Briefly, the study population was defined as patients undergoing minimally invasive mitral valve operations with all possible indications, using all available approaches and materials. The MMIR database was designed specifically to assess patients with mitral valve disease and patients undergoing mini mitral valve surgery. It includes variables on clinical data, risk assessment variables, surgery related data, perioperative outcomes, echocardiographic data and long-term outcomes. All centers provided data by using the same definitions and assessment measures according to the current European Society of Cardiology or ACC/AHA/HRS Guidelines, Euroscore II model and Mitral Valve Academy Research Consortium endpoint definitions [4]. The completed data forms were forwarded by the participating MMIR sites to the coordinating center at The Polytechnic University of Marche and reviewed for face validity and completeness. Definitions of endpoints were as follows: low cardiac output: inotropic support >24 h or the use of temporary mechanical circulatory support. Acute kidney injury: the maximal change in serum creatinine (sCr) from baseline to 7 days post-procedure as follows: (1) stage 1, increase in sCr to 150%–199%, increase of ≥ 0.3 mg/dl (≥ 26.4 mmol/l) within 48 h, or urine output < 0.5 ml/kg/h for ≥ 6 h but < 12 h, (2) stage 2, increase in sCr to 200%–299% or urine output < 0.5 ml/kg/h for ≥ 12 h but < 24 h and (3) stage 3, increase in sCr to ≥ 300%, sCr of ≥ 4.0 mg/dl (≥ 354 mmol/l) with an acute increase of ≥ 0.5 mg/dl (44 mmol/l), urine output < 0.3 ml/kg/h for >24 h, or anuria for ≥ 12 h or patients receiving renal replacement therapy. Stroke: duration of a focal or global neurological deficit ≥ 24 h or < 24 h if available neuroimaging documents a new intracranial or subarachnoid hemorrhage or central nervous system infarction or the neurological deficit results in death. Residual MR: mild, moderate or severe MR (postoperative echo). Outcomes of valvular surgery was recorded based on information from discharge echocardiography. Technical success was defined according to MVARC criteria: absence of procedural mortality, successful access, correct positioning of the first intended device, and freedom from emergency surgery or reintervention related to the device or access procedure.

At the time of this study 7,513 consecutive patients were enrolled in the registry, encompassing all consecutive MICS cases in the centers between 2015 and 2021. Of these, 6,463 who underwent first time mitral with or without tricuspid valve surgery were analysed and subdivided into three groups. Direct vision, where surgery was performed with a headlight and no further video-support for the case, video-assisted, where an endoscope was used to support direct vision, and fully endoscopic, where the surgeon was guided only by viusualization on a screen (2d or 3D). Exclusion criteria were reoperation, partial sternotomy access, concomitant aortic valve or root replacement. The study protocol was approved by the local institutional review board of all centers based on the approval of the coordinating center (n.2020189, July 30th, 2020), and consent of patients were obtained when required.

### Statistical analysis

Categorical data are expressed as frequencies and percentages; continuous data are presented as mean ± SD or median with first and third quartiles (Q1–Q3) as appropriate. Missing data were not defaulted to zero, and denominators reflect only actual reported data.

Multivariable logistic regression models were developed to evaluate the association between surgical approaches and outcomes, adjusting for potential confounders (age, sex, diabetes, chronic lung disease, dialysis, peripheral arteriopathy, cerebrovascular arteriopathy, obesity, urgent status, NYHA (III/IV), low EF (< 50), atrial fibrillation, concomitant tricuspid regurgitation, enrolling center). The models were checked for multi-collinearity, and goodness-of-fit was assessed. A two-sided p-value of less than 0.05 was considered statistically significant. Analyses were performed using Statistical Package for Social Sciences, version 29.0 (IBM SPSS Inc., Chicago, IL, USA).

## Results

Table 1 shows the demographic data of the patients that were subdivided into three groups (direct vision, video-assisted, fully endoscopic). There were 56 robotic cases in the entire population, which were youngest, most selected and had no mortality. These case were not further considered for this analysis.

**Table 1:** Demographic data

	Direct vision (*n* = 1594) *n* (%)	Video-assisted (*n* = 2850) *N* (%)	Fully-endoscopic (*n* = 1963) *n* (%)	*P* value
Male	918 (57.6)	1619 (57.1)	1210 (61.6)	0.002
Age, median (IQR)	66 (57–74)	64 (53–72)	64 (53–73)	< 0.001
NYHA III-IV	689 (51.3)	1227 (43.3)	875 (44.8)	< 0.001
Hypertension	1036 (69.4)	1465 (51.5)	839 (54.3)	< 0.001
Diabetes	207 (13)	168 (5.9)	130 (6.6)	< 0.001
Smoking	220 (14.8)	353 (12.4)	178 (11.5)	0.004
Obesity	301 (20.2)	337 (12.3)	236 (12)	< 0.001
Atrial fibrillation	591 (37.1)	1010 (35.5)	524 (34.5)	0.2
Pacemaker	98 (6.2)	29 (1)	35 (1.8)	< 0.001
Dialysis	32 (2)	14 (0.5)	12 (0.6)	< 0.001
CAD	290 (20.3)	288 (10.1)	212 (10.8)	< 0.001
Chronic lung disease	130 (8.2)	231 (8.1)	139 (7.1)	0.2
Active endocarditis	51 (3.2)	103 (3.7)	60 (3.1)	0.3
Cerebrovascular arteriopathy	32 (2.3)	46 (1.6)	24 (1.2)	0.09
Peripheral arteriopathy	40 (2.5)	43 (1.5)	64 (3.3)	< 0.001
Pulmonary hypertension	708 (44.8)	934 (36.4)	802 (40.9)	< 0.001
MV disease aetiology				< 0.001
*Degenerative*	977 (72.3)	1819 (68.3)	1488 (76.1)	
*Functional*	225 (16.6)	365 (13.7)	284 (14.5)	
*Rheumatic*	59 (4.4)	285 (10.7)	94 (4.8)	
*Other*	117 (7.3)	91 (3.2)	67 (3.4)	
MV regurgitation (moderate-severe)	1428 (89.6)	2685 (94.3)	1896 (96.6)	< 0.001
MV stenosis (moderate-severe)	71 (4.6)	263 (9.3)	88 (5.7)	< 0.001
Tricuspid regurgitation (moderate-severe)	374 (23.6)	763 (27.3)	502 (25.6)	< 0.001
LVEF < 50%	339 (22)	450 (16.0)	264 (13.5)	< 0.001
Urgent/emergent status	179 (11.2)	71 (2.5)	110 (5.6)	< 0.001
ES II, median (IQR)	1.5 (0.8–2.8)	1.1 (0.7–1.9)	1.2 (0.8–2.2)	< 0.001

ES: EuroSCORE. IQR: interquartile range. LVEF: left ventricular ejection fraction. MV: mitral valve. NYHA: New York Heart Association, CPB: cardio-pulmonary bypass.AF: atrial fibrillation

In the three groups used for comparison, average age was 65 years with the oldest patients in the direct vision group. This group also had more patients in NYHA class III and IV, more diabetes and obesity, more preoperative pacemakers, more peripheral artery disease, the most patients with ejection fractions below 50% and the lowest rate of severe mitral regurgitation as indication for surgery. As a result, the EuroSCORE II was highest in the direct vision group. However, all differences were rather small.

Table 2 shows the operative characteristics in the three groups. The highest rate of repair was observed in the fully endoscopic group, with mitral valve replacement in 11% versus 23% in the other groups. Concomitant tricuspid surgery was performed in 20% of the video-assisted and direct vision groups and only 13% in the fully endoscopic group. Despite the lower rate of tricuspid surgery, both cardiopulmonary bypass and cross-clamp times were highest in the fully endoscopic group. Cross-clamp time in the direct vision group was more than 20 min shorter than in the video-assisted or fully endoscopic group. Technical success of surgery was highest in the direct vision group with 99.3%, but was also very high in the other two groups (above 96%).

**Table 2:** Intra-operative outcome

	Direct vision (*n* = 1594) *n* (%)	Video-assisted (*n* = 2850) *N* (%)	Fully-endoscopic (*n* = 1963) *n* (%)	*P* value
Arterial cannulation site				< 0.001
Femoral artery	1438 (96.9)	2716 (96.9)	1936 (98.6)	
Axillary artery	10 (0.7)	76 (2.7)	24 (1.2)	
Ascending aorta	9 (0.6)	10 (0.4)	3 (0.2)	
Other	27 (1.8)	-	-	
Myocardial protection				0.003
*Cardioplegia*	1589 (99.7)	2793 (99.7)	1942 (98.9)	
*Ventricular* *fibrillation*	1 (0.1)	9 (0.3)	19 (1)	
*Beating heart*	4 (0.3)	-	1 (0.1)	
Cardioplegia type				< 0.001
*Blood*	363 (22.8)	1393 (50.4)	745 (38)	
*Crystalloid*	1222 (76.9)	1367 (49.4)	1191 (60.8)	
Type of surgery				< 0.001
*Mitral valve repair*	1209 (75.8)	2141 (75.1)	1727 (88)	
*Mitral valve* *replacement*	369 (23.1)	667 (23.4)	222 (11.3)	
*Replacement due to* *unsuccessful repair*	16 (1)	42 (1.5)	13 (0.7)	
Mitral valve Repair				
*Annuloplasty Ring*	1049 (98.9)	2108 (98.7)	1709 (98.9)	< 0.001
*Resection*	61 (6.2)	478 (22.8)	249 (14.4)	< 0.001
*Sliding plasty*	30 (3)	75 (3.6)	17 (0.9)	< 0.001
*Artificial chords*	645 (56.9)	1268 (60.6)	1169 (67.7)	< 0.001
*Edge to edge*	48 (4.2)	55 (2.6)	23 (1.3)	< 0.001
Concomitant Tricuspid surgery	342 (21.5)	399 (20)	259 (13.3)	< 0.001
Concomitant Atrial Fibrillation surgery	287 (18)	494 (17.3)	412 (21)	< 0.001
Repeated x-clamping	24 (1.7)	83 (3)	45 (2.3)	0.07
CPB time (minutes), median (IQR)	120 (98.8–145.3.8.3)	130 (98–170)	145 (119–182)	< 0.001
X-Clamp time (minutes), median (IQR)	65 (52–84)	87 (65–113)	90 (71–113)	< 0.001
Technical success	1365 (99.3)	2653 (96.2)	1880 (97.6)	< 0.001

CPB: Cardio-Pulmonary Bypass; IQR: InterQuartile Range

Table 3 shows perioperative outcomes. In-hospital mortality and stroke rates were low in all three groups and not significantly different. Intubation times were lowest in the direct vision and highest in the video-assisted group. Low cardiac output tended to be higher in the direct vision group, which may potentially berelated to the higher fraction of patients with low ejection fraction. Acute kidney injury was numerically highest in the fully endoscopic group and lowest in the video-assisted group. However, this difference was not significant.

**Table 3:** Peri-operative outcomes

	Direct vision (*n* = 1594) *n* (%)	Video-assisted (*n* = 2850) *N* (%)	Fully-endoscopic (*n* = 1963) *n* (%)	*P* value
In-hospital mortality	22 (1.4)	34 (1.2)	26 (1.3)	0.7
30-days mortality	27 (2.2)	38 (1.9)	29 (1.7)	0.4
Stroke	16 (1)	40 (1.4)	22 (1.1)	0.7
Delirium	152 (11)	161 (5.8)	64 (4.1)	< 0.001
Intubation time (hours), median (IQR)	5 (3–8)	9.4 (6–14)	7.7 (5–13)	< 0.001
Ventilation > 24 h	96 (6.1)	255 (9)	121 (8)	0.001
Bleeding	88 (6.4)	141 (5)	115 (5.9)	0.2
New onset AF	205 (12.9)	458 (16.3)	256 (16.9)	0.004
Myocardial infarction	14 (0.9)	25 (0.9)	15 (0.8)	0.9
*Periprocedural (≤48 h)*	12 (0.8)	20 (0.7)	15 (0.8)	
*Spontaneous (> 48 h)*	2 (0.1)	5 (0.2)	-	
Low cardiac output	83 (5.6)	88 (3.2)	47 (2.4)	< 0.001
Acute kidney injury	89 (6.5)	147 (5.2)	109 (7.2)	0.07
Postoperative dialysis	27 (2)	28 (1)	19 (1.2)	0.06
Vascular complications	13 (0.9)	60 (2.1)	35 (2)	0.001
*Major*	3 (0.2)	42 (1.5)	30 (1.7)	
*Minor*	10 (0.7)	18 (0.6)	5 (0.3)	
Wound complications	66 (4.1)	97 (3.5)	75 (3.8)	0.2
ICU stay (hours), median (IQR)	29 (7–77)	24 (20–46)	21 (18–26)	< 0.001
Hospital stay (days), median (IQR)	9 (6–14)	8 (6–11)	7 (6–9)	< 0.001

IQR: InterQuartile Range

Table 4 shows outcomes reported after adjusting for potential confounders with the video-assisted group used as comparator. Direct vision had the lowest ventilation times and wound complications but most patients with low cardiac output and longer length of stay. The fully endoscopic approach did not significantly affect any outcomes compared to the video-assisted approach.

**Table 4:** Adjusted models for in-hospital outcomes

Unadjusted analysis				Adjusted analysis*		
	OR^1^	95% CI^1^	*p*-value	OR^1^	95% CI^1^	*p*-value
**30 day Mortality**						
Surgical approach categories						
Direct vision	0.85	0.45, 1.60	0.6	1.16	0.67, 1.99	0.6
Video-assisted (ref)	**—**	**—**	**—**	**—**	**—**	**—**
Fully-endoscopic	0.84	0.44, 1.58	0.6	1.11	0.66, 1.85	0.7
**Stroke**						
Surgical approach categories						
Direct vision	0.77	0.39, 1.49	0.4	0.71	0.39, 1.27	0.3
Video-assisted (ref)	**—**	**—**	**—**	**—**	**—**	**—**
Fully-endoscopic	0.83	0.46, 1.49	0.5	0.79	0.47, 1.34	0.4
**MACCE**						
Surgical approach categories						
Direct vision	0.87	0.58, 1.31	0.5	0.95	0.67, 1.36	0.8
Video-assisted (ref)	**—**	**—**	**—**	**—**	**—**	**—**
Fully-endoscopic	0.73	0.49, 1.11	0.1	0.85	0.60, 1.19	0.3
**Low cardiac Output**						
Surgical approach categories						
Direct vision	**1.99**	**1.43**,** 2.80**	**0.001**	**1.83**	**1.34**,** 2.49**	**0.001**
Video-assisted (ref)	—	—	—	—	—	—
Fully-endoscopic	0.92	0.62, 1.36	0.7	0.75	0.53, 1.08	0.1
**Ventilation > 24 h**						
Surgical approach categories						
**Direct vision**	**0.66**	**0.52**,** 0.84**	**0.001**	**0.52**	**0.39**,** 0.69**	**0.001**
Video-assisted (ref)	—	—	—	—	—	—
Fully-endoscopic	0.88	0.71, 1.11	0.3	0.91	0.71, 1.16	0.4
**Composite Wound Complications (Thoracic**,** Groin)**						
Surgical approach categories						
**Direct vision**	**0.59**	**0.39**,** 0.89**	**0.01**	**0.61**	**0.39**,** 0.93**	**0.02**
Video-assisted (ref)	—	—	—	—	—	—
Fully-endoscopic	1.11	0.81, 1.51	0.5	1.30	0.94, 1.80	0.1
	Standardized ß	95% CI	p-value	Standardized ß	95% CI	p-value

CI = Confidence Interval. OR = Odds Ratio. **Bold** = significant (p<0.05)

**Fig. 1:**
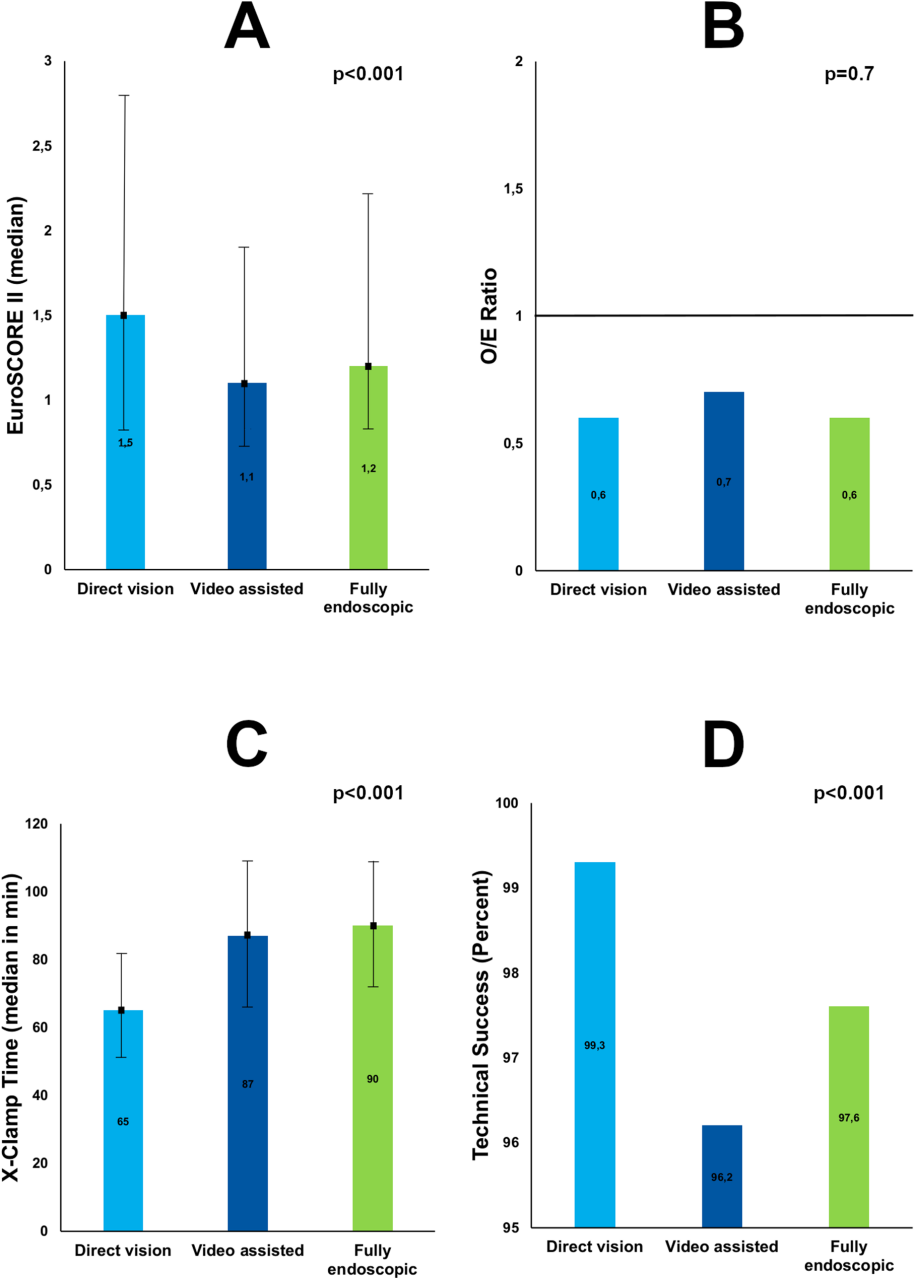
Comparison of key characteristics (**A**: EuroScore II), outcomes (**B**: O/E ratio) and technical features (**C**: X-clamp time, **D**: Technical Success) of minimally-invasive mitral with or without tricuspid surgery through a direct vision, a video-assisted or a fully endoscopic approach from the international mini-mitral registry

Figure 1 shows a comparison of EuroSCORE II, O/E ratio, cross-clamp time and technical success. The figure illustrates the similarity of the outcomes and also shows the excellent outcomes in all groups with O/E ratios well below 1 with all three approaches. Given the large number of observations, one may suggest that there is a trend towards higher risk in the direct vision group with shorter cross-clamp times and higher rates of technical success.

## Discussion

In this large registry, the type of minimally-invasive approach did not significantly affect outcome. It appears that fully endoscopic approach is used more selectively. Mastering both techniques may optimize patient care. In general, this and previous reports of the MMIVR may set a bar for standards of minimally-invasive mitral surgery [2, 4].

The findings in this retrospective registry analysis resemble the recently published outcomes from the Mini-Mitral-Trial, where minimally-invasive mitral valve surgery did not significantly affect perioperative or long-term outcomes in mitral valve surgery [5]. While in the Mini-Mitral trial there was a difference in quality-of-life at six weeks, the difference was gone after three months [5]. From this perspective, it may not be surprising that there are no big differences between different minimally-invasive approaches. However, inspecting the data carefully appears to reveal certain patterns.

First, current publications and discussions in various different meeting scenarios often promote the use of 3D endoscopic or robotic approaches for mitral valve surgery [1, 6–10]. Arguments such as better visualization, better teaching abilities and greater degrees of freedom and instrument use are brought forward to support these more technically challenging and in the case of robotics more personnel and more cost intensive approaches [11, 12]. While these approaches usually do not end up with incisions that are smaller than direct vision (in the hand of direct vision experts), the omission of rib spreading and limitations of the extent of the thoracic cage opening (rib space opening) may indeed be an advantage that may be associated with lower degrees of pain and postoperative complications. A recent analysis assessed robotic and non-robotic mitral valve surgery with respect to pain perception [13]. The investigators demonstrated that there was less opioid requirements in robotic cases. However, the amount of pain perception in patients was identical. The number of robotic cases in the mini-mitral registry was too small (*n* = 56) to be meaningfully included in this analysis. The results of this analysis suggests that adding an endoscope to minimally-invasive cardiac surgery does not improve patient outcomes. This recognition may be very important, specifically for surgeons who begin minimally-invasive mitral surgery, since changing approach, instruments and the type of vision at the same time may exceed a feasible amount of change to allow the maintenance of safe procedure performance.

This rationale is also supported by assessing the cardiopulmonary bypass and cross-clamp times. It appears that adding an endoscope adds time to the duration of both cardiopulmonary bypass use and cross-clamping. Given the fact that cross-clamp time correlates with mortality and the onset of acute kidney injury [2, 14], this relationship should not be forgotten when surgically approaching the heart in a new way. It is interesting to note that the outcomes in the fully endoscopic group were not better than the other two groups, although more isolated mitral valve cases were located here and the amount of patients with low ejection fraction was lowest. It is also interesting that similar outcomes in this more selected group correlated with the longest cardiopulmonary bypass and clamp times and also the numerically highest rate of acute kidney injury postoperatively. However, it is important to state that these associations may not reflect causal relationships. It isalso important to state that the influence of individual surgical skill cannot be assessed by these data, as surgeon identifiers were not collected by the registry.

Weighing the outcomes against each other reveals another important aspect. Consider an equal distribution of patient complexity for the participating expert centers in the mini-mitral registry. If some cases (e.g., robotic or fully endoscopic) appear more selected than endoscopic-assisted or direct vision, how are patients treated who do not qualify for the fully endoscopic approach. If the operating surgeons focus on the endoscopic minimally-invasive approach only, greater fractions of all patients will receive sternotomy. However, we did not collect the information on sternotomy mitral valve surgery in the participating centers for the same time period and can therefore not answer this question. In any case, mastering both techniques may therefore improve the penetration of minimally-invasive approaches to the mitral valve and therefore patient care.

## Limitations

This analysis is of course limited by its retrospective nature. That means that certain complications that may occur only long-term may not have been recorded in this registry. For instance, direct vision usually requires rib spreading, which entails the possibility of postoperative lung herniation and may inflict more pain, which was not specifically addressed in the registry. Although extremely rare, this complication is almost impossible to occur with fully endoscopic or robotic cases, if the opening of the intercostal space is indeed kept as small as the skin incision. Furthermore, many aspects in patient selection and individual operative competence may have influenced the outcomes.

## Conclusion

We demonstrate in this large mini-mitral registry analysis that the type of minimally-invasive approach does not appear to significantly affect outcome. The data suggest that fully endoscopic are used more selectively. Mastering both techniques may optimize patient care.

## Data Availability

The datasets used and/or analysed during the current study are available from the corresponding author on reasonable request.
